# Tm–guided exon–exon junction RT-PCR enables specific detection of RNA variants lacking easily distinguishable exonic regions

**DOI:** 10.64898/2026.04.02.716213

**Published:** 2026-04-05

**Authors:** Junyeop Ahn, Donald J Zack, Ping-Wu Zhang

**Affiliations:** 1. Department of Ophthalmology, Stem Cell Ocular Regenerative Medicine Center, Wilmer Eye Institute, Johns Hopkins University School of Medicine; Baltimore, MD, 21231, USA; 2. Solomon H. Snyder Department of Neuroscience, Department of Molecular Biology and Genetics, Department of Genetic Medicine, Center for Nanomedicine at the Wilmer Eye Institute, The Johns Hopkins University School of Medicine, Baltimore, Maryland, United States

**Keywords:** Transcript-specific PCR, RNA variant, detection, heteroduplex, heteroquadruplex, exon–exon junction, primer design

## Abstract

Accurate detection of RNA splice variants is often hindered when transcripts lack large distinguishable exonic regions, making conventional PCR strategies challenging. We developed a simple melting temperature (Tm)-guided exon–exon junction (EEJ) RT-PCR method to enable variant-specific detection under these conditions. Uni-directional primers spanning exon–exon junctions were designed so that approximately each half anneals to adjacent exons. The Tm of each half-site was set >7°C below the annealing temperature, preventing stable binding to individual exons and enforcing junction-dependent amplification. The method was evaluated using HTRA1-AS1 long noncoding RNA variants that share overlapping exon sequences but differ in splice connectivity. HTRA1-AS1 comprises five variants, only one with a large distinguishable exon. Tm-guided EEJ primers robustly discriminated the remaining four variants. After optimization, amplification yielded sharp, single bands with minimal cross-reactivity. Compared with conventional designs, this approach reduced heteroduplex and heteroquadruplex formation, improving band clarity. Sanger sequencing confirmed junction specificity, and the method performed well in multiplex settings.

Overall, Tm-guided EEJ RT-PCR is a cost-effective, high-resolution approach for detecting RNA variants lacking easily distinguishable exonic regions, readily compatible with standard RT-PCR and qPCR workflows.

## Introduction

Alternative splicing is an important mechanism that expands transcriptomic and proteomic diversity in eukaryotic organisms. Human genes have average of eight exons and three or more alternatively spliced mRNA isoforms. It is estimated that more than 90% of multi-exon human genes generate multiple transcript variants through alternative exon usage, exon skipping, alternative splice sites, and intron retention. These variants can differ in regulatory function, subcellular localization, and protein-coding potential, and often play critical roles in development, disease progression, and cellular responses to stress [1–3].

Reliable detection and quantification of specific transcript variants remain technically challenging, particularly when transcripts share extensive exon overlap. Conventional RT-PCR and quantitative PCR approaches typically rely on primers targeting large distinguishable exons to individual transcripts. However, for transcript variants that lack these regions and instead share very similar exon sequences, differing only in exon connectivity generated by alternative splicing, design of exon-targeting primer that distinguish highly similar isoforms, leading to ambiguous detection or co-amplification of multiple transcripts [4, 5]. High-throughput sequencing approaches, such as long-read sequencing technologies, can effectively identify and distinguish highly similar splice isoforms [6,7]. However, these methods are costly, computationally intensive, and not always practical for routine validation or targeted transcript analysis.

A cost-effective PCR-based alternative strategy for distinguishing highly similar transcript variants is to target exon–exon junctions generated during RNA splicing. Because each transcript variant is defined by a specific combination of exon junctions, primers spanning these junctions can provide variant specificity even in the absence of large distinguishable exonic regions. Intron-spanning primers have been widely used to reduce genomic DNA amplification and to detect splice variants in qPCR assays [8,9]. In addition, computational tools such as Ex-Ex Primer and ExonSurfer have been developed to assist in exon–exon junction primer design for transcript detection and genomic DNA avoidance [10,11]. However, experimentally validated strategies and details for using exon–exon junction primers to distinguish transcript variants lacking large distinguishable exonic regions have not been well established.

In this study, we present a practical melting temperature (Tm)–guided exon–exon junction RT-PCR method for the identification and discrimination of RNA transcript variants that share largely identical exonic sequences. This approach utilizes a dual-recognition primer design in which each primer spans a splice junction, with the Tm of each half-site optimized to prevent stable annealing to individual exons. Consequently, efficient amplification depends on correct exon–exon connectivity, conferring high specificity while minimizing nonspecific amplification and heteroduplex formation. Here we outline the primer design principles, optimization strategy, and experimental validation using RT-PCR and Sanger sequencing. Overall, this method provides a simple, robust, and broadly applicable strategy for detecting closely related transcript variants in studies of alternative splicing and transcript-specific gene expression.

## Methods

### Primer design

Primer pairs were designed to selectively amplify transcript variants based on exon–exon junctions rather than exon-specific sequences. Primers were initially designed using Primer3plus software (https://www.primer3plus.com/index.html), and exon–exon junction primers were further refined manually. For each target transcript, either the forward or reverse primer was designed to span the exon–exon junction, with approximately half of the primer sequence complementary to the upstream exon and the remaining portion complementary to the downstream exon. This design ensures that amplification occurs only when the specific exon junction is present in the cDNA template, thereby preventing amplification from transcripts lacking the junction or from genomic DNA containing introns.

Primer lengths were typically 18–24 nucleotides, with melting temperatures (Tm) of approximately 58–64 °C and GC content between 40–60%. When sequence constraints limited GC content, primer length was adjusted to maintain appropriate Tm. Amplicon sizes were designed to range from 150 to 250 bp to ensure efficient amplification and compatibility with both conventional RT-PCR and quantitative PCR. Primer specificity was evaluated using BLAST against the reference genome (UCSC Genome Browser, USA) and transcriptome to minimize off-target amplification. When multiple transcript variants shared similar junctions, additional primers targeting alternative junctions were designed to improve discrimination. All primers were synthesized by Integrated DNA Technologies (IDT, USA).

### PCR amplification and optimization

Primer melting temperatures were calculated using the NEB Tm Calculator (v1.16.10) with Phusion High-Fidelity DNA Polymerase (HF buffer conditions) as the reference. The Tm values of the 5′-end and 3′-end segments of each junction-spanning primer were calculated separately, along with the overall primer Tm, to guide primer design and PCR optimization.

PCR amplification was performed using Phusion High-Fidelity PCR Master Mix (Thermo Fisher Scientific, USA) or PyroMark PCR Master Mix (Qiagen, USA) according to the manufacturers’ instructions. PCR conditions were optimized to ensure that the partial Tm values of junction-spanning primers remained below the annealing temperature, thereby minimizing nonspecific amplification.

### Sanger sequencing validation

PCR products corresponding to each transcript variant were validated by Sanger sequencing at the Johns Hopkins Genetic Resources Core Facility. PCR products were submitted without purification and sequenced using standard protocols. Sequencing chromatograms were analyzed and aligned to reference sequences to confirm the identity of the amplified products. This validation step verified that each amplicon corresponded precisely to the intended exon–exon junction, confirming the specificity and reliability of the primer design and amplification strategy.

## Results

### Melting temperature–guided exon–exon junction primer design enables RNA variant–specific RT-PCR

**Figure 1.**
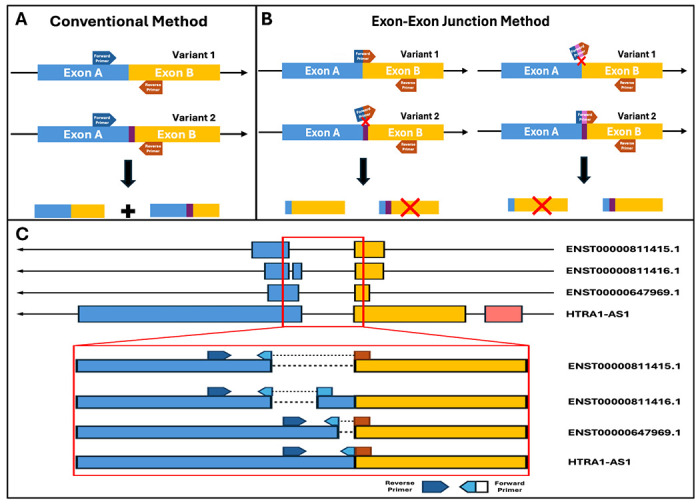
Exon–exon junction primers enable transcript variant–specific detection. **(A)** Conventional method: Primers designed within shared exons (Exon A and Exon B) amplify multiple transcript variants due to identical exon sequences, resulting in non-specific detection. **(B)** Exon–exon junction method: A junction-spanning primer is designed across the exon–exon boundary. Amplification occurs only when the correct exon combination is present, enabling selective detection of the target variant, while mismatched variants are not amplified. **(C)** Application to *HTRA1-AS1* four transcript variants: Schematic of multiple variants lacking exons. Junction-specific primer design targets distinct exon connectivity (highlighted region), allowing discrimination of closely related transcript variants. The inset illustrates primer positioning across exon–exon junctions for variant-specific amplification.

To enable transcript variant–specific detection in cases where variants lack large distinguishable exonic regions, we developed a uni-directional primer design strategy based on a dual-recognition mechanism at exon–exon junctions (Figure 1). In this approach, a single primer (forward or reverse) spans a splice junction, with approximately half of its sequence complementary to the upstream exon and the remaining half complementary to the downstream exon. This configuration requires simultaneous annealing to both exons in the correct adjacency, thereby conferring junction-level specificity.

A key feature of this strategy is the thermodynamic discrimination between partial and full primer binding. The melting temperature (Tm) of each half-primer segment was deliberately designed to be lower than the PCR annealing temperature (typically 58–64 °C), generally by >7 °C. Under these conditions, partial annealing to a single exon is thermodynamically unstable and does not support productive extension. In contrast, when the primer encounters the correct exon–exon junction, both halves anneal cooperatively to form a stable duplex, enabling efficient amplification. This design suppresses amplification from transcripts sharing only a single exon as well as from genomic DNA containing intronic sequences.

As illustrated in Figure 1A, conventional primers targeting shared exons (Exon A and Exon B) amplify multiple transcript variants due to identical target exon sequences, resulting in poor specificity. In contrast, the exon–exon junction strategy (Figure 1B) restricts amplification to transcripts containing the precise exon combination recognized by the junction-spanning primer. Variants lacking the correct exon connectivity fail to support full primer annealing and are therefore not amplified, enabling clear discrimination among closely related transcripts.

We applied this strategy to the lncRNA HTRA1-AS1 locus, which contains multiple transcript variants with highly overlapping exon structures but distinct exon connectivity patterns (Figure 1C). Because these variants lack large distinguishable exonic regions, conventional approaches cannot distinguish them. By designing primers targeting junctions to each splice configuration, we achieved selective amplification of individual variants. The inset in Figure 1C highlights the positioning of junction-spanning primers, illustrating how variant specificity is achieved through differential exon pairing.

Further optimization focused on balancing primer composition to minimize nonspecific extension. Increasing sequence contribution toward the 5′ region improved specificity by reducing spurious priming from the 3′ end, which is critical for polymerase extension. Through optimization, we established design parameters that maintain both specificity and amplification efficiency.

Using these principles, we generated a panel of uni-directional exon–exon junction primers targeting distinct splice junctions. Four forward primers were designed to recognize different exon–exon boundaries and paired with a common reverse primer located in a shared downstream exon. This modular configuration enables scalable detection in which amplification is strictly dependent on correct exon adjacency. Consequently, only transcript variants containing the targeted junction are efficiently amplified, whereas variants sharing partial sequence identity are excluded.

The calculated melting temperatures for each primer half and the full-length primers are summarized in Table 1, providing quantitative support for the thermodynamic basis of the design.

### PCR polymerase selection and PCR optimization

PCR polymerase selection was critical for achieving robust and specific amplification using junction-spanning primers. We evaluated several commonly used PCR polymerases under identical cycling conditions and observed substantial differences in amplification efficiency and specificity. High-fidelity polymerases with optimized buffer systems consistently produced stronger and more reproducible products, whereas some enzymes yielded weak bands or exhibited nonspecific amplification. Notably, performance differences were evident even between similar Phusion High-Fidelity PCR Master Mix formulations obtained from different commercial suppliers. Based on these comparisons, Thermo Fisher Phusion Flash High-Fidelity PCR Master Mix and Qiagen 2× PyroMark PCR Master Mix (containing HotStarTaq DNA polymerase and 3 mM Mg^2+^) were selected for subsequent experiments. Both systems generated clear, specific bands with minimal background, demonstrating their suitability for exon–exon junction PCR (data for other polymerases not shown).

These findings underscore the importance of polymerase selection for the reliable detection of transcript variants lacking large distinguishable exonic regions. While predicted melting temperatures (Tm) provide a useful starting point for primer design and initial annealing temperature selection, the optimal annealing temperature must be determined empirically. Annealing conditions have a strong impact on assay performance and are also dependent on the PCR polymerase used. For example, the optimal annealing temperature for Phusion Flash High-Fidelity PCR Master Mix differs from that of PyroMark PCR Master Mix (Supplementary Data 1–3).

### Validation of variant-specific amplification by Sanger sequencing

**Figure 2.**
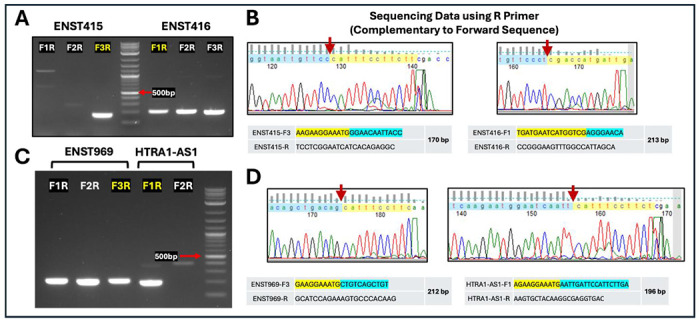
Variant-specific amplification of *HTRA1-AS1* transcripts using exon–exon junction primers. **(A, C)** Agarose gel electrophoresis showing PCR products generated with different primer combinations (F1R, F2R, F3R). Clear bands at the expected sizes (~170–213 bp) are observed only for the correct variant, with minimal non-specific amplification. DNA ladders are included for size reference, with the ~500 bp band indicated. **(B, D)** Sanger sequencing confirms that the PCR products correspond to the expected exon–exon junction sequences. Red arrows mark the precise junction sites. Yellow and blue highlighted regions indicate the primer-binding sequences (including reverse-complement alignment), demonstrating accurate primer–template pairing and variant-specific amplification. ENST415, ENST416, and ENST969 are abbreviated identifiers for the lncRNA transcript variants ENST0000811415.1, ENST0000811416.1, and ENST0000647969.1, respectively

Using the optimized PCR conditions, each exon–exon junction primer set generated a single, discrete band at the expected size for its corresponding transcript variant (Figure 2A, C). No additional bands or smearing were observed, indicating high amplification specificity and minimal nonspecific products. Importantly, primer sets targeting different splice junctions produced mutually exclusive amplification patterns across variants, consistent with junction-dependent recognition. The observed amplicon sizes (~170–213 bp) matched the predicted lengths based on primer design and exon connectivity, further supporting accurate target amplification.

To rigorously validate specificity at the sequence level, PCR products were directly subjected to Sanger sequencing without gel purification, providing a stringent test of amplification fidelity. Sequencing chromatograms obtained using reverse primers (complementary to the forward strand) exhibited clean, high-quality peaks with minimal background noise (Figure 2B, D). Critically, the sequences spanning the amplified regions precisely matched the designed exon–exon junctions, with the junction boundaries clearly identifiable at the expected positions (indicated by arrows). No evidence of mixed sequencing signals or off-target amplification was detected, indicating that a single dominant product was generated in each reaction.

For all tested transcript variants (ENST415, ENST416, ENST969, and *HTRA1-AS1*), the sequencing results confirmed that amplification occurred only when the correct exon adjacency was present, consistent with the double-recognition mechanism of the uni-directional junction primers. Variants lacking the targeted exon–exon junction did not yield detectable products, demonstrating effective discrimination among closely related variants that share common exon sequences. Only the ENST415-specific forward primer F3, when paired with the common reverse primer (F3R), produced a specific PCR band after optimization, similar to the *HTRA1-AS1* forward primer F1 combined with the common reverse primer (F1R). In contrast, all three primer pairs performed efficiently for ENST416 and ENST969.

### Exon–exon junction RT-PCR reduces heteroduplex and quadruplex formation in the simultaneous detection of multiple gene transcripts

**Figure 3.**
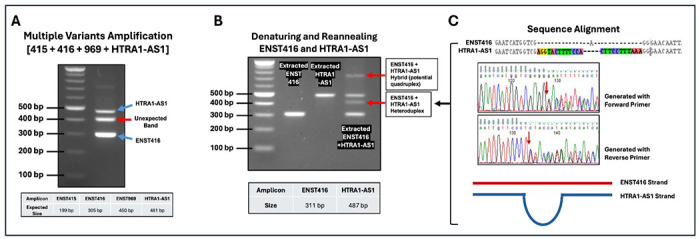
Heteroduplex and quadruplex formation during detection of RNA variants using conventional RT-PCR. (**A**) Co-amplification of four HTRA1-AS1 variants (ENST415, ENST416, ENST969, and HTRA1-AS1) using a common primer set produces multiple bands on agarose gel electrophoresis, including expected products and an unexpected intermediate band (red arrow), suggesting nonspecific hybridization. (**B**) Validation of heteroduplex formation by denaturation and reannealing. After denaturation and reannealing single bands together, which individually ENST416 and HTRA1-AS1 products, additional higher-molecular-weight bands (red arrows) were generated, consistent with heteroduplex formation between the two variants. Amplicon sizes are indicated below. (**C**) Sequence alignment and Sanger sequencing traces of the heteroduplex product generated using forward and reverse primers. Overlapping chromatogram peaks and misaligned base calls confirm the presence of hybrid DNA strands derived from ENST416 and HTRA1-AS1. A schematic model illustrates heteroduplex formation through partial base pairing between homologous regions of the two transcripts.

Four HTRA1-AS1 variants (ENST415, ENST416, ENST969, and HTRA1-AS1) were initially co-amplified using a common primer set to assess amplification specificity. Agarose gel electrophoresis revealed three bands corresponding to ENST416 (305bp), HTRA1-AS1 (481bp), and an unexpected intermediate band (about 400bp) between them (Figure 3A). Sanger sequencing of the gel-extracted intermediate band confirmed that it represented a heteroduplex composed of hybrid strands derived from ENST416 and HTRA1-AS1 (Figure 3C).

To exclude the possibility that this band resulted from co-migrating independent amplicons, gel purified ENST416 and HTRA1-AS1 PCR products were subjected to denaturation (98 °C) followed by reannealing (63 °C). Individual samples produced a single band, whereas the mixed sample generated an additional band (~400 bp), consistent with heteroduplex formation between the two variants and even a higher molecular weight heteroquadruplex band (Figure 3B).

**Figure 4.**
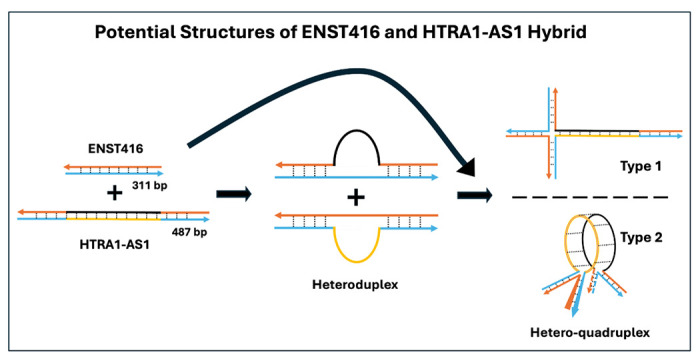
Potential hybrid structures formed between ENST416 and HTRA1-AS1 PCR products. Schematic illustrating the formation of hybrid DNA structures between ENST416 (311 bp) and HTRA1-AS1 (487 bp) amplicons containing partially homologous regions. Upon denaturation and re-annealing, the shared sequences enable intermolecular base pairing, leading to heteroduplex formation with single-stranded loop regions corresponding to non-homologous segments. Two possible higher-order structures are proposed: (Type 1) a cruciform-like heteroduplex formed through crosswise pairing of homologous regions, and (Type 2) a hetero-quadruplex-like assembly involving multistrand interactions and circularized pairing of complementary segments. Arrows indicate strand orientation, and dashed lines represent base-paired regions. These structures may contribute to heteroduplex DNA artifacts observed during PCR amplification.

As illustrated in Figure 4, partial sequence homology between amplicons of different lengths promotes the formation of heteroduplex and higher-order structures (quadruplex) during re-annealing. PCR products or these intermediates can further assemble into higher-order structures, including loop-containing quadruplexes and multi-stranded quadruplexes, which contribute to aberrant bands observed on agarose gels. The presence of unpaired regions and overlapping homologous segments facilitates these interactions, highlighting how shared sequence regions between PCR products drive heteroduplex and quadruplex formation.

**Figure 5.**
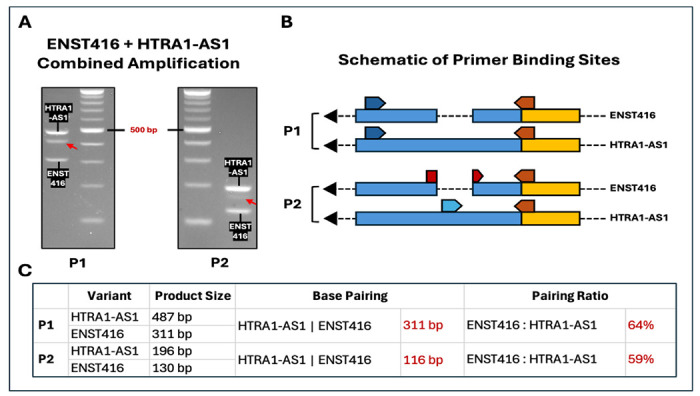
Exon–exon junction RT-PCR reduces heteroduplex formation. (**A–C**) Agarose gel electrophoresis showing co-amplification of ENST416 and HTRA1-AS1 using different primer designs. (**A**) Conventional primer set (P1) generates strong heteroduplex formation, evident as an additional band between the expected amplicons. (**B**) Use of variant-specific reverse primers (P2) reduces heteroduplex formation, although intermediate bands remain detectable. (**C**) Exon–exon junction–targeted primer design (P3) markedly suppresses heteroduplex formation, yielding predominantly distinct bands corresponding to each variant. Amplicon sizes and shared sequence lengths between variants are indicated below each panel. (**D**) Schematic representation of primer design strategies and their effects on heteroduplex formation. P1 employs shared primer binding regions, promoting hybridization between partially homologous amplicons. P2 introduces variant-specific primers to reduce overlap. P3 incorporates an ENST416-specific exon–exon junction forward primer, which limits extension from mismatched templates and disrupts hybrid strand formation. The reduced sequence complementarity and junction-specific targeting together minimize heteroduplex formation.

We next evaluated strategies to reduce heteroduplex or quadruplex formation. An additional primer set (P1) was used to selectively co-amplify ENST416 and HTRA1-AS1 variants, resulting in the formation of heteroduplex products alongside the two variant amplicons. To minimize extensive homologous regions, a second primer configuration (P2) was designed using a common forward primer and two distinct reverse primers—including an exon–exon junction primer—that uniquely anneal to each variant (Supplementary Data 2). This design excludes the potential for loop formation between variants. Heteroduplex band was still observed with the P2 primer combination; however, their intensity was reduced (Figure 5).

Thus, exon–exon junction RT-PCR provides greater flexibility in primer design, enabling reduction of heteroduplex formation by limiting amplification of homologous regions between variants (Figure 5A–C).

Collectively, these results demonstrate that heteroduplex formation in conventional RT-PCR arises from partial sequence complementarity between co-amplified variants and can be substantially minimized by exon–exon junction–targeted primer design. This uni-directional EEJ strategy enables highly specific, sequence-validated detection of transcript variants, even when conventional exon-targeting approaches fail to resolve closely related splice isoforms.

## Discussion

Accurate detection of transcript variants is essential for understanding gene regulation and transcript-specific biological functions. Alternative splicing greatly expands transcriptomic diversity in eukaryotic genomes, with most human genes generating multiple RNA variants [1–3]. However, many variants share highly similar exon sequences and differ only in exon connectivity, making variant-specific detection challenging using conventional PCR strategies that rely on exon-targeting primers [5]. In addition to limited specificity, co-amplification of closely related transcripts frequently leads to heteroduplex DNA formation, complicating gel interpretation and reducing assay reliability.

In this study, we present a simple and effective PCR-based approach that distinguishes transcript variants by targeting exon–exon junctions unique to individual splice configurations. Because splice junctions are generated only after RNA processing, primers spanning these junctions enable selective amplification of specific variants even in the absence of large distinguishable exonic regions. This strategy achieves transcript-level specificity using standard RT-PCR or qPCR workflows [5,9].

A central feature of this method is the use of T_m_-guided, uni-directional exon–exon junction primers, in which approximately half of the primer sequence anneals to each of two adjacent exons. By designing each half-primer with a melting temperature lower than the PCR annealing temperature, partial annealing to a single exon is thermodynamically unstable. Efficient amplification therefore occurs only when both halves simultaneously bind across the correct exon boundary. This double-recognition mechanism enhances specificity, minimizes cross-amplification, and functionally suppresses extension from mismatched templates.

Importantly, this thermodynamic constraint also reduces heteroduplex and quadruplex DNA formation. Conventional RT-PCR often generates heteroduplex and quadruplex products when partially homologous amplicons reanneal, resulting in intermediate bands. In contrast, the EEJ primer design limits the production of overlapping amplicons and reduces effective cross-hybridization including heteroduplex and non-G-heteroquadruplex between variants, thereby improving band clarity and interpretability [12,13]. This effect likely reflects a reduction in the length and/or proportion of homologous pairing regions (Figure 5A, 5C). Consistent with this, our experiments show that junction-targeted primers markedly decrease heteroduplex formation compared with conventional primer designs, particularly in multiplex settings involving closely related transcripts.

The EEJ method is experimentally accessible and readily adaptable. Primer design follows standard PCR principles, and assay validation can be performed using routine RT-PCR and Sanger sequencing. Compared with transcriptome-wide approaches such as long-read RNA sequencing, this strategy is cost-effective and requires minimal computational resources and can be easily implemented in most molecular biology laboratories [6,7,14,15]. Thus, exon–exon junction RT-PCR provides a practical tool for validating transcript structures predicted by RNA-seq and for monitoring specific splice variants under defined experimental conditions.

Several considerations are important for optimal performance. First, accurate annotation of exon–exon junctions is essential for primer design. Second, PCR polymerase selection and reaction optimization can significantly influence amplification efficiency across splice junctions. Third, transcript variants that share identical exon–exon junctions cannot be distinguished by this approach alone and may require complementary methods, such as long-read sequencing, length-dependent PCR, or rapid amplification of cDNA ends (RACE) [14,15].

In summary, the T_m_-guided exon–exon junction primer strategy provides a robust, specific, and experimentally straightforward approach for distinguishing closely related RNA variants. By enabling variant-specific detection without reliance on large distinguishable exonic regions and by minimizing heteroduplex formation, this method expands the experimental toolkit for studying alternative splicing and transcript diversity, particularly in complex loci such as long noncoding RNAs [16,17].

## Supplementary Material

Supplement 1

## Figures and Tables

**Table 1:** Exon-Exon junction Primers and their Tm values

Primer Name	Sequence (5′ | 3′)	Ratio	Product Size	Partial Tm (°C)	Tota I Tm (°C)	GC %
ENST415-F1	TGCACGAAGAAGGAAATGGGAAC	18:5	176 bp	55 | -*	63	48
ENST415-F2	CGAAGAAGGAAATGGGAACAATA	14:9	172 bp	42 | 15	58	39
ENST415-F3	AAGAAGGAAATGGGAACAAKACC	12:12	170 bp	32 | 34	59	38
ENST415-R	TCCTCGGAATCATCACAGAGGC				64	55
ENST416-F1	TGATGAATCATGGTCGAGGGAACA	16:8	213 bp	49 | 17	64	46
ENST416-F2	TGATGAATCATGGTCGAGGGAACAAn	16:11	213 bp	49 | 31	65	41
ENST416-F3	AATCATGGTCGAGGGAACAATTACC	11:14	208 bp	34 | 43	63	44
ENST416-R	CCGGGAAGTTTGGCCATTAGCA				65	55
ENST969-F1	AGAAGGAAATGCTGTCAGCTGTCT	11:13	213 bp	29 | 45	64	46
ENST969-F2	AGGAAATGCTGTCAGCTGTCTG	8:14	210 bp	10 | 48	63	50
ENST969-F3	GAAGGAAATGCTGTCAGCTGT	10:11	212 bp	24 | 38	61	48
ENST969-R	GCATCCAGAAAGTGCCCACAAG				64	55
HTRAl-ASl-Fl	AGAAGGAAATGAATTGATTCCATTCTTGA	11:18	196 bp	29 | 48	62	31
HTRA1-AS1-F2	ACGAAGAAGGAAATGAATTGATTCCA	15:11	200 bp	45 | 28	61	35
HTRAl-ASl-R	AAGTGCTACAAGGCGAGGTGAC				65	55

Note: Primer sequences are listed in the 5′→3′ direction. Forward primers (F1–F3) span exon–exon junctions, with ratios indicating the nucleotide contributions from each exon. Product size denotes the expected amplicon length. Partial Tm refers to the melting temperature of each exon-matching segment, while total Tm indicates the overall primer melting temperature. GC% represents GC content. Reverse primers (R) are shared within each transcript group.

“*” indicates that the Tm is not available because the primer length is shorter than the minimal functional length.

ENST415, ENST416, and ENST969 are abbreviations for ENST00000811415.1, ENST00000811416.1, and ENST00000647969.1, respectively

## References

[1] Pan Q, Shai O, Lee LJ, Frey BJ, Blencowe BJ. Deep surveying of alternative splicing complexity in the human transcriptome by high-throughput sequencing. Nat Genet. 2008;40(12):1413–1415. 18978789 10.1038/ng.259

[2] Wang ET, Sandberg R, Luo S, Khrebtukova I, Zhang L, Mayr C, Alternative variant regulation in human tissue transcriptomes. Nature. 2008;456(7221):470–476. 18978772 10.1038/nature07509 PMC2593745

[3] Lee Y, Rio DC. Mechanisms and regulation of alternative pre-mRNA splicing. Annu Rev Biochem. 2015; 84:291–323. 25784052 10.1146/annurev-biochem-060614-034316 PMC4526142

[4] Shila S, Dahiya V, Hisle C, Bahadursingh E, Thiyagarajan R, Fields PE, Rumi MAK. mRNA Isoforms and Variants in Health and Disease. Int J Mol Sci. 2025 Sep 25;26(19):9356. doi: 10.3390/ijms26199356. 41096623 PMC12524470

[5] Vo K, Shila S, Sharma Y, Pei GJ, Rosales CY, Dahiya V, Fields PE, Rumi MAK. Detection of mRNA Transcript Variants. Genes (Basel). 2025 Mar 16;16(3):343. doi: 10.3390/genes16030343. 40149494 PMC11942493

[6] Ozsolak F, Milos PM. RNA sequencing: advances, challenges and opportunities. Nat Rev Genet. 2011;12(2):87–98. 21191423 10.1038/nrg2934 PMC3031867

[7] Stark R, Grzelak M, Hadfield J. RNA sequencing: the teenage years. Nat Rev Genet. 2019;20(11):631–656. 31341269 10.1038/s41576-019-0150-2

[8] Thornton B, Basu C. Real-time PCR (qPCR) primer design using free online software. Biochem Mol Biol Educ. 2011;39(2):145–154. 21445907 10.1002/bmb.20461

[9] Nolan T, Hands RE, Bustin SA. Quantification of mRNA using real-time RT-PCR. Nat Protoc. 2006;1(3):1559–1582. 17406449 10.1038/nprot.2006.236

[10] Monfort-Lanzas P, Rusu EC, Parrakova L, Karg CA, Kernbichler DE, Rieder D, Lackner P, Hackl H, Gostner JM. ExonSurfer: a web-tool to design primers at exon-exon junctions. BMC Genomics. 2024 Jun 12;25(1):594. doi: 10.1186/s12864-024-10456-2. 38867172 PMC11170769

[11] Govindkumar B, Kavyashree B, Patel K, Sasidharan K, Siva Arumugam T, Thomas L, Praveena BKG, Raksha HN, Menon R, Acharya KK. Ex-Ex Primer: An experimentally validated tool for designing oligonucleotides spanning spliced nucleic acid regions from multiple species. J Biotechnol. 2022 Jan 10; 343:1–6. doi: 10.1016/j.jbiotec.2021.10.009. Epub 2021 Oct 29. 34756973

[12] Tsai YC, Qi H, Lin CP, Lin RK, Kerrigan JE, Rzuczek SG, LaVoie EJ, Rice JE, Pilch DS, Lyu YL, Liu LF. A G-quadruplex stabilizer induces M-phase cell cycle arrest. J Biol Chem. 2009 Aug 21;284(34):22535–43. doi: 10.1074/jbc.M109.020230. Epub 2009 Jun 16. 19531483 PMC2755660

[13] Liu S, Peng P, Wang H, Shi L, Li T. Thioflavin T binds dimeric parallel-stranded GA-containing non-G-quadruplex DNAs: a general approach to lighting up double-stranded scaffolds. Nucleic Acids Res. 2017 Dec 1;45(21):12080–12089. doi: 10.1093/nar/gkx942. 29059300 PMC5716147

[14] Tilgner H, Grubert F, Sharon D, Snyder MP. Defining a personal, allele-specific, and single-molecule long-read transcriptome. Proc Natl Acad Sci U S A. 2014;111(27):9869–9874. 24961374 10.1073/pnas.1400447111 PMC4103364

[15] Byrne A, Beaudin AE, Olsen HE, Jain M, Cole C, Palmer T, Nanopore long-read RNA sequencing reveals widespread transcriptional variation among the surface receptors of individual B cells. Nat Commun. 2017; 8:16027. 28722025 10.1038/ncomms16027 PMC5524981

[16] Mercer TR, Dinger ME, Mattick JS. Long non-coding RNAs: insights into functions. Nat Rev Genet. 2009;10(3):155–159. 19188922 10.1038/nrg2521

[17] Derrien T, Johnson R, Bussotti G, Tanzer A, Djebali S, Tilgner H, The GENCODE v7 catalog of human long non-coding RNAs: analysis of their gene structure, evolution, and expression. Genome Res. 2012;22(9):1775–1789. 22955988 10.1101/gr.132159.111 PMC3431493

