# Supplementary material for: Tm–guided exon–exon junction RT-PCR enables specific detection of RNA variants lacking easily distinguishable exonic regions
Source: bioRxiv. 2026 Apr 5:2026.04.02.716213. Preprint. [Version 1] doi: 10.64898/2026.04.02.716213 (PMC13060199; doi:10.64898/2026.04.02.716213)
Supplement: Supplement 1 [file media-1.pdf]

Supplementary Data

1. Variant-specific PCR Primer Information

Variant	Primer Name	Sequence (5' | 3')	Ratio	Product Size		Partial Tm (°C)	Total Tm (°C)	GC %	Result
ENST415	ENST415-F1	TGCACGAAGAAGGAAATGGGAAC	18:5	176 bp		55 | -*	63	48	
	ENST415-F2	CGAAGAAGGAAATGGGAACAATA	14:10	172 bp		42 | 15	58	39	
	ENST415-F3	AAGAAGGAAATGGGAACAATTACC	12:12	170 bp		32 | 34	59	38	Worked
	ENST415-R	TCCTCGGAATCATCACAGAGGC					64	55	
ENST416	ENST416-F1	TGATGAATCATGGTCGAGGGAACA	16:8	213 bp		49 | 17	64	46	Worked
	ENST416-F2	TGATGAATCATGGTCGAGGGAACAATT	16:11	213 bp		49 | 31	65	41	Worked
	ENST416-F3	AATCATGGTCGAGGGAACAATTACC	11:14	208 bp		34 | 43	63	44	Worked
	ENST416-R	CCGGGAAGTTTGGCCATTAGCA					65	55	
ENST969	ENST969-F1	AGAAGGAAATGCTGTCAGCTGTCT	11:13	213 bp		29 | 45	64	46	Worked
	ENST969-F2	AGGAAATGCTGTCAGCTGTCTG	8:14	210 bp		10 | 48	63	50	Worked
	ENST969-F3	GAAGGAAATGCTGTCAGCTGT	10:11	212 bp		24 | 38	61	48	Worked
	ENST969-R	GCATCCAGAAAGTGCCCACAAG					64	55	
HTRA1-AS1	HTRA1-AS1-F1	AGAAGGAAATGAATTGATTCCATTCTTGA	11:18	196 bp		29 | 48	62	31	Worked
	HTRA1-AS1-F2	ACGAAGAAGGAAATGAATTGATTCCA	15:11	200 bp		45 | 28	61	35	
	HTRA1-AS1-R	AAGTGCTACAAGGCGAGGTGAC					65	55	
ENST416	**Primer Name**	**Sequence (5' | 5' | 3')**	**Ratio**	**Product Size**		**Partial Tm (°C)**	**Total Tm (°C)**	**GC %**	
	HTRA1-AS1-F1	AGAAGGAAATGAATTGATTCCATTCTTGA	11:18			29 | 48	62	31	
	ENST416-cross-R	GGTAATTGTTCCCTCGACCATGA	14:9	130 bp		43 | 27	48	62	Worked

2. Variants Combined PCR Primer Information

Primer Pair	Primer Name	Sequence (5' | 3')	Ratio	Product Size		Partial Tm (°C)	Total Tm (°C)	GC %
				ENST416	HTRA1-AS1			
P1	HTRA1-AS1-F1	AGAAGGAAATGAATTGATTCCATTCTTGA	11:18			29 | 48	62	31
	ENST416-R	CCGGGAAGTTTGGCCATTAGCA		305 bp	487 bp		65	55
P2	HTRA1-AS1-F1	AGAAGGAAATGAATTGATTCCATTCTTGA	11:18			29 | 48	62	31
	ENST416-cross-R	GGTAATTGTTCCCTCGACCATGA	14:9	130 bp		43 | 27	48	62
	HTRA1-AS1-R	AAGTGCTACAAGGCGAGGTGAC			196 bp		65	55

Primer Name	Sequence (5' | 3')	Product Size				Total Tm (°C)	GC %
		ENST415	ENST416	ENST969	HTRA1-AS1		
Multi-Var-F	GTGTGCCTACGTGTGTCCATCA	199 bp	305 bp	450 bp	481 bp	64	55
Multi-Var-R	CGGAATCATCACAGAGGCTGGG					65	59

3. PCR Protocol and Reagent Information

	Reagent	PCR Cycle	T_a (°C)
**Variant-specific PCR**			
ENST415	Phusion Flash HF	36	65
ENST416	Phusion Flash HF	36	65
ENST969	PyroMark	44	62
HTRA1-AS1	PyroMark	44	62
**ENST416 and HTRA1-AS1 Combined PCR**			
P1	PyroMark	40	63
P2	PyroMark	40	63
P3	PyroMark	40	60
**ENST415, ENST416, ENST969 and HTRA1-AS1 Combined PCR**			
Multi-Var	Pyromark	40	63
